# 

**Published:** 1842-10-01

**Authors:** 


**CONTENTS OF No. 40**

Dr. Waters' Case of Anomalous Periodical Ovarian Tumour, - - 625
BIBLIOGRAPHICAL NOTICE: Dr. Robert Liston's Elements of Surgery, - 627
EDITORIAL: Dr. Hayward's Correction, - - - - - - - - 628
Medical Coroner, - - - - - - - - - - 628
The late Baron Larrey, - - - - - - - - 629
Miscellaneous Notices, - - - - - - - - 630
ANALECTA: Dr. Fischer on Belladonna in Strangulated Hernia, - - 631
Dr. Giraldes on Organs for the Secretion of Sweat, - - 632
Dr. Mackay on the Adulteration of the Cantharis Vesicatoria, 632
Typhus Fever in Paris, - - - - - - - - 633
M. Velpeau on Cysts of the Eylid, - - - - - - 633
Enlarged Prostate Mistaken for Calculus, - - - - - 634
Devilliers and Chailly on Auscultation in Pregnancy, - - 634
Wardleworth, on Black Pitch in the treatment of Hæmorrhoids, 635
Signor Della Fanteria on the reunion of two fingers, &c. - 637
Dr. Green's Observations on the treatment of Eczema, - 637
Post-mortem examination of a soldier 102 years old, - - 640

